# Can stem cells really regenerate the human heart? Use your noggin, dickkopf! Lessons from developmental biology

**DOI:** 10.5830/CVJA-2013-045

**Published:** 2013-06

**Authors:** Paula Sommer

**Affiliations:** School of Life Sciences, University of KwaZulu-Natal, Durban, South Africa

**Keywords:** heart development, stem cell clinical trials, iPS cells, ES cells, paracrine signalling

## Abstract

**Abstract:**

The human heart is the first organ to develop and its development is fairly well characterised. In theory, the heart has the capacity to regenerate, as its cardiomyocytes may be capable of cell division and the adult heart contains a cardiac stem cell niche, presumably capable of differentiating into cardiomyocytes and other cardiac-associated cell types. However, as with most other organs, these mechanisms are not activated upon serious injury. Several experimental options to induce regeneration of the damaged heart tissue are available: activate the endogenous cardiomyocytes to divide, coax the endogenous population of stem cells to divide and differentiate, or add exogenous cell-based therapy to replace the lost cardiac tissue. This review is a summary of the recent research into all these avenues, discussing the reasons for the limited successes of clinical trials using stem cells after cardiac injury and explaining new advances in basic science. It concludes with a reiteration that chances of successful regeneration would be improved by understanding and implementing the basics of heart development and stem cell biology.

## Development of the heart

Human development begins with the fertilisation of an ovum by a sperm, initiating furious but intricately controlled cell division. At implantation, the embryo is known as a blastocyst, consisting of an outer trophoblast and an inner cell mass composed of embryonic stem (ES) cells. These ES cells divide, move, respond to cues from themselves and each other, and demonstrate pluripotency, the ability to develop into all the tissues and organs of the human body.

As the embryo develops into a foetus, the cells lose pluripotency and progress towards a more differentiated state, contributing to the formation of organs. Their potency therefore becomes more and more restricted. Every organ and tissue in the adult body retains a niche of stem cells whose potency is generally specific to the cells of the resident tissue.

The heart is the first organ to form in the developing embryo. The vertebrate heart develops from two regions of splanchnic mesoderm, one on each side of the developing embryo, that interact with the directly adjacent tissue, the anterior endoderm. The presence of the anterior endoderm is essential for heart development, and the interaction between these two tissues results in the specification of cells destined to form the heart – the cardiogenic mesoderm.^1^ The presence of certain cardiac-restricted transcription factors such as *Gata4*^2^ and *Nkx2-5*^3^ are essential for restricting the mesoderm to a cardiac fate.

The anterior endoderm secretes factors such as bone morphogenetic proteins (BMPs) and fibroblast growth factors (FGFs). The *Bmp2* gene particularly plays a role as, in mice in which the *Bmp2* gene has been knocked out (*Bmp2*^-/-^ mice), the heart either does not develop or develops poorly.^4^ This range of different phenotypes suggests a degree of genetic redundancy, where other BMPs compensate for the lack of *Bmp2*. Such effects are also noted for the different FGFs.

Along with the positive signals initiating heart development, inhibitory signals prevent the heart from forming where it shouldn’t. The notochord, which serves to define the central axis of the embryo, secretes the BMP inhibitors noggin and chordin, preventing the heart from forming in the centre of the embryo. The anterior endoderm secretes Wnt inhibitors such as cerebrus, dickkopf and crescent, which prevent Wnts from binding to their receptors. Therefore cardiac precursor cells are specified in the places where BMPs (from the lateral mesoderm and endoderm) and Wnt antagonists (from the anterior endoderm) coincide.^5^

This simplified description of heart development serves to show that there is no straightforward recipe for the development of the human heart. There is, however, information that can be exploited.

***Key message:*** Heart development is a complex process promoted by positive signals such as BMPs and shaped by negative signals such as the Wnt inhibitors, cerebrus and dickkopf, and the BMP inhibitors, noggin and chordin.

## Can the human heart be induced to regenerate after injury?

An estimated 17 million people worldwide die annually from cardiovascular disease, particularly heart attacks and strokes (http://www.who.int/cardiovascular_diseases/resources/atlas/en/). Cardiovascular disease is also prevalent in South Africa, resulting in 195 deaths per day between 1997 and 2004 (http://www.mrc.ac.za/chronic/heartandstroke.pdf).

The major cause of heart failure is the death of cardiomyocytes, where a typical large myocardial infarct (MI) kills around one billion myocytes (one-quarter of the heart).^6^ The current treatments do not address the problem of the reduced pool of cardiomyocytes but rather involve transplantation or insertion of mechanical ventricular assist devices.

For many years, prevailing dogma insisted that the heart was a static post-mitotic organ incapable of regeneration. While heart tissue has shown a capacity to regenerate, there is intense controversy over whether cardiomyocyte division plays a role in regeneration. Some *in vivo* studies have shown evidence of possible cardiomyoctye division, although they fail to agree on the rate of cardiomyocyte turnover,^7^,^8^ and have been heavily criticised for their methodology.^9^ Regardless, it is evident that their possible ability to divide does not extend to repairing extensively damaged heart tissue.

The heart has also been shown to harbour a compartment of multi-potent cardiac stem cells and other progenitor cells that can differentiate into myocytes and coronary vessels. Again, there has been much controversy surrounding this discovery. Some believe that new myocytes may arise from the de-differentiation of mature myocytes back to their immature state, allowing them to acquire an immature phenotype and therefore to divide.^10^

There are those that query whether the identified cardiac stem cell population is fully distinct from haematopoetic stem cells (HSCs) in the bone marrow, as these cells are able to enter the circulation, home to organs and trans-differentiate, acquiring a myocyte lineage.^11^ This was initially a surprising finding as only embryonic stem cells are pluripotent, and as they contribute to the development of tissues, their potency becomes more and more restricted to cells of that tissue.

It is thought that commitment to a developmental fate is irreversible but plasticity has been shown, particularly with HSCs. This line of thought has been heavily criticised, with studies showing that HSCs cannot trans-differentiate into cardiomyocytes after MI.^12^,^13^

The existence of a c-kit^+^ population of cardiac stem cells able to self-renew and to differentiate into cardiomyocytes, smooth muscle and endothelial cells has been demonstrated.^14^ Detractors argue against the existence of these cells, reasoning that spontaneous repair after injury does not occur. However, stem cell niches have been described in many organs and while these cells have been shown to play a role in regulating tissue homeostasis, many do not effectively respond to aging or injury, possibly because the adult environment is not permissible.

***Key message:*** Several experimental options to induce regeneration of damaged heart tissue require investigation: activation of the endogenous populations of cardiomyocytes and/or stem cells, or the addition of exogenous cell-based therapy to replace lost cardiac tissue.

## Exogenous cell-based therapy: the different types of stem cells used in clinical trials for heart regeneration after injury

There are currently 30 to 40 registered clinical trials using different types of stem cells to treat various types of cardiovascular disease (http://www.clinicaltrials.gov/; www.clinicaltrialsregister.eu^15^). The overwhelming majority of the registered trials, completed, on-going or not yet recruiting, involve the use of stem cells derived from the bone marrow. The bone marrow is an attractive source of stem cells as the cells can be obtained relatively easily. The bone marrow contains a hetergoneous population of stem cells of various lineages (including the blood mononuclear cells, B-cells, T-cells and monocytes, as well as rare progenitor cells such as haematopoietic stem cells, mesenchymal stem cells, endothelial progenitor cells, CD_34_^+^ and CD_133_^+^ cells).^16^

The bone marrow stem cell fraction can either be administered whole or distinct bone marrow cell populations can be isolated on the basis of specific cellular markers. Approximately half the registered trials use whole bone marrow fractions while the others use specific cells purified, using specific markers, from the bone marrow fraction. These include bone marrow mononuclear cells, bone marrow-derived mesenchymal stem cells, endothelial cells, CD_34_^+^ and CD_133_^+^ cells.

Treatment with G-CSF (granulocyte colony-stimulating factor) stimulates the movement of bone marrow stem cells into the bloodstream and has been used in trials of patients suffering from cardiovascular disease, either as the sole treatment to incite movement of bone marrow stem cells into the bloodstream or in conjunction with administration of stem cells. Trials have been performed with both autologous and allogeneic bone marrow stem cells. The majority of trials using bone marrow stem cells use autologous bone marrow stem cells, whole or purified.

Although cardiac stem cells are more difficult to isolate, as they can only be harvested from endomyocardial biopsies and require careful growth conditions and identification using markers such as c-kit, Sca-1 and Isl-1, trials have also been performed with autologous cardiac stem cells. One of these trials is slightly more complex and involves the addition of cardiac stem cells along with a bFGF gel mat during coronary artery bypass surgery for local release of bFGF.^17^ As there are those who are concerned about the ‘stemness’ of cardiac stem cells (the ability of these cells to form cardiac tissue), there is a trial using autologous cardiospheres. When cardiac stem cells derived from biopsies are allowed to grow *in vitro*, the cells form spheres, hence cardiospheres, and are presumably more committed to a cardiac stem cell fate.

***Key message:*** Both bone marrow and cardiac-derived stem cells have been used or are currently being used in clinical trials to determine whether these cells could contribute to cardiac repair.

## Does exogenous administration of autologous or allogeneic stem cells aid in cardiac repair?

Results from randomised trials using bone marrow mononuclear cells demonstrated modest cell therapy-mediated improvements in ventricular function.^9^ In one of the largest studies to date, 204 randomised patients diagnosed with acute MI received intracoronary delivery of bone marrow cells or vehicle control. After one year these patients showed significant improvements in cardiac function.^18^ A two-year follow up revealed that these positive effects were preserved.^19^

The authors showed experimentally that less than 20% of the administered stem cells were retained in the heart,^20^ which indicates that some cells do home in on the target tissue, but it also shows that only a few cells are needed to exert positive effects. These results are in contrast to those obtained by a similarly constructed study where patients suffering from acute ST-segment elevation MI were administered bone marrow-derived stem cells. While initial results were promising,^21^ the 18-month and five-year follow ups have shown that, while the treatment had an overall positive effect, this was attributed to the early improvement, with little sustained effect seen.^22^

The general consensus is that bone marrow stem cell treatment moderately improves heart function, does not decrease mortality or morbidity significantly in long-term follow up, and has, as yet, not been associated with any significant safety concerns.^23^ How these cells act to repair the damaged tissue is not known. Some believe that the bone marrow cells trans-differentiate into cardiomyocytes.^10^ However, it is generally believed that these cells secrete paracrine factors, creating a permissible environment, which stimulates the endogenous cardiomyocyte progenitor cells or adult cardiomyocytes to divide. While this is entirely probable, there is little evidence for this.

Similarly, trials using selected cell populations such as endothelial progenitor cells (which promote angiogenesis and possibly secrete paracrine factors that may promote cardiomyocyte division) or mesenchymal stem cells (which can differentiate into cardiomyocytes although the rate of differentiation is low) isolated from bone marrow have shown similar results to the whole bone marrow fraction studies described.^9^

One of the first trials, the Stem Cell Infusion in Patients with Ischemic cardiOmyopathy or SCIPIO trial, using purified cardiac stem cells, has recently produced interim results. In this study, autologous c-kit+ cells were isolated and grown from tissue harvested during coronary artery bypass surgery, and administered to the patient at a later stage by intracoronary infusion. Cardiac magnetic resonance showed that, not only is the procedure feasible, but also that administration of the cells produced a striking improvement in both global and regional left ventricular function, a reduction in infarct size, and an increase in viable tissue, which persisted at least one year after administration, consistent with cardiac regeneration.^24^,^25^

Initial results from the CADUCEUS (CArdiosphere-Derived aUtologous stem CElls to reverse ventricUlar dySfunction) randomised, controlled trial have also been released. Here, after myocardial infarction, autologous cardiac stem cells were isolated from endomyocardial biopsy specimens and allowed to grow *in vitro* to form cardiospheres. These were then administered via intracoronary delivery. Initial results indicate that the process is safe and viable, and showed a decrease in scar tissue mass after MI, and regeneration of viable myocardium.^26^

It is possible that the positive results of the cardiac stem cells and cardiosphere trials are not due to direct stem cell action,^27^ as the cardiac ‘stemness’ of cardiac stem cells and cardiospheres has been questioned. Using an elegant labelling technique where transgenic mice were generated with enhanced green fluorescent protein under the control of the c-kit gene promoter, a report showed that, while c-kit marks cardiac progenitor cells during development, in the adult, it is not a marker for cells capable of cardiomyogenesis. This study shows that, at least in this experimental model, cardiac c-kit^+^ cells^28^ and cardiospehere-derived cells lack cardiomyogenic potential.^29^

All of these described trials were designed with the intent that the stem cells, whether bone marrow or cardiac derived, engraft into the diseased tissue and differentiate into functional cardiomyocytes. However, the results suggest that it is not that simple and that the modest improvements seen are probably due to the exogenous cells creating a permissible environment (by secreting factors and appropriate signals) that induces the endogenous cardiomyocytes or cardiac stem cells to proliferate. It is therefore suggested that basic knowledge of stem cell and developmental biology be exploited to further increase the positive effects seen thus far.^30^

***Key message:*** Bone marrow or cardiac-derived stem cells do contribute to cardiac repair or survival, however, it seems that these cells do not contribute directly. Their action appears to be effected by paracrine secretions, which create a permissible environment for the endogenous repair mechanisms.

## Exploitation of developmental and stem cell biology knowledge

The clinical trials described so far have had only marginal, if any, success. This may be due to the use of adult stem cells that lack the necessary plasticity to differentiate into cardiomyocytes. It may be that more highly plastic cells are needed to recapitulate cardiomyogenesis. In theory, this requirement should be satisfied by the use of cardiac stem cells, cells that should be able to differentiate into cardiomyocytes. However, it appears that they may not persist into adulthood or may express different markers in the adult. Their use is also complicated by the fact that autologous cardiac stem cells are harvested after damage to the heart and, generally, from older patients.

The most plastic or potent cells are ES cells. ES cells were first isolated from a human embryo in 1998 and since then, several ES cell lines have been created, which perpetuate in culture. These cells cannot simply be administered to patients, as their intrinsic potency and ability to divide causes teratomas, even several years after therapeutic delivery.

These issues can be overcome by directing human ES cells along a pathway of differentiation towards a cardiomyocyte lineage. Various recipes involving sequential exposure to BMP2, FGF and Wnt, and BMP inhibitors as in normal heart development have been shown to generate cells with the potential to form cardiomyocytes, smooth muscle and endothelial cells. When transplanted into primates, these cells did not form teratomas but contributed to the repair of scar tissue. The rationale behind this basic work is to generate a pool of pure ES-derived cardiomyocytes, which could be used for ‘off-the-shelf’ therapy.^31^ This is a clever but very expensive approach that is still muddied by the initial use of ethically controversial ES cells.

Induced pluripotent stem (iPS) cells are adult, somatic cells which have been re-programmed by the addition of three or four embryonic transcription factors to become pluripotent. These cells overcome the ethical concerns created by embryonic stem cells and also graft rejection, as they can be engineered from a patient’s own stromal cells for autologous transplantation.

Mouse embryonic fibroblasts were induced to become pluripotent by forced expression of the pluripotency markers, OCT3/4, SOX2, KLF4 and c-MYC. These iPS cells were administered to athymic nude mice after the induction of myocardial ischaemia by left coronary artery ligation. These cells did not form tumours and successfully integrated into the allogeneic host heart parenchyma, contributing to tissue reconstruction with synchronised cardiovasculogenesis.^32^

These initial studies in animal models are promising, however re-programming involves using viruses to introduce the pluripotency factors into the cells, creating concerns. The elimination of the need for viruses to re-programme the cells would make iPS cells a very attractive option for the generation of cardiomyocytes.

Another option is simply to skip the induction of pluripotency and directly re-programme somatic cells into functional cardiomyocytes. Research has shown that cardiomyocytelike cells can be derived from directly re-programming postnatal dermal or cardiac fibroblasts, using three developmental transcription factors: *Gata4*, *Mef2c* and *Tbx5*.^33^ Indeed, these cells have been shown in mouse models of MI to contribute to decrease in infarct size. As with the iPS cells, however, the use of viruses to re-programme the cells still prevents translation into clinical trials.

While all of these options are possible if the procedures are made virus free, perhaps the simplest option would be to circumvent cell-based therapy. All the clinical trial evidence thus far points to the fact that the stem cells create a permissible environment via paracrine signalling that serves to amplify the endogenous regenerative response and/or stimulate cell cycle re-entry of endogenous cardiomyocytes. Adult stem cells, particularly mesenchymal stem cells, have been shown to secrete a wide variety of factors that promote protection of the myocardia, neovascularisation, cardiac remodelling, and improve contractility.^34^ The use of these molecules alone as a treatment to stimulate endogenous cardiac repair is an exciting and promising avenue of research.

***Key message:*** Cardiomyocytes can be synthetically created from ES, iPS or even by simple trans-differentiation of somatic cells using developmental cues. However, the creation of these cells involves the use of viruses, which pose a risk to patients. Paracrine factors are postulated to contribute to the positive results seen in clinical trials. Identification of the secreted molecules could pave the way for cell-free mechanisms of stimulating the endogenous repair mechanism.

## Conclusions

There are numerous on-going or completed clinical trials to assess the abilities of bone marrow or cardiac-derived stem cells to regenerate cardiac tissue destroyed by cardiovascular disease. These trials have been shown to have limited success. It appears that, although the initial aims of these trials were that the exogenous stem cells would directly contribute to cardiac repair by integrating into the target tissue, proliferating and differentiating into cardiac-associated cell types, the positive action exerted by these cells may be indirect.

It is believed that stem cells secrete various growth factors, cytokines and signalling molecules that stimulate the endogenous stem cells or cardiomyocytes to proliferate. The inability of the exogenously administered stem cells to contribute directly may be due to their lack of potency. It is therefore thought that, to regenerate substantial cardiac tissue, either synthetically generated stem cells such as iPS or directly re-programmed somatic cells (generated without the use of viral vectors) are a more feasible, though expensive, option.

It is clear that understanding of the molecules that direct heart development and the environment in which the heart develops, and the use of this information in creating new treatments is essential. The development of a ‘cell-free’ treatment with the administration of molecules that either stimulate the endogenous cardiomyocytes, or stem cells to divide, or molecules that create a permissive environment to stimulate regeneration would be an ideal solution, eliminating the need for surgery.
